# Crosstalk between mismatch repair and base excision repair in human gastric cancer

**DOI:** 10.18632/oncotarget.10185

**Published:** 2016-06-20

**Authors:** Valeria Simonelli, Giuseppe Leuzzi, Giorgia Basile, Mariarosaria D’Errico, Paola Fortini, Annapaola Franchitto, Valentina Viti, Ashley R. Brown, Eleonora Parlanti, Barbara Pascucci, Domenico Palli, Alessandro Giuliani, Fabio Palombo, Robert W. Sobol, Eugenia Dogliotti

**Affiliations:** ^1^ Department of Environment and Primary Prevention, Istituto Superiore di Sanità, Rome, Italy; ^2^ Istituto di Ricerche Biologia Molecolare P. Angeletti (IRBM), Pomezia (Rome), Italy; ^3^ University of Pittsburgh Cancer Institute, Hillman Cancer Center, Pittsburgh, PA, USA; ^4^ Institute of Cristallography, Consiglio Nazionale delle Ricerche, Monterotondo Stazione, Rome, Italy; ^5^ Molecular and Nutritional Epidemiology Unit, CSPO, Scientific Institute of Tuscany, Florence, Italy; ^6^ Takis Biotech, Castel Romano, Rome, Italy; ^7^ Department of Pharmacology and Chemical Biology, University of Pittsburgh, Pittsburgh, PA, USA; ^8^ Department of Oncologic Sciences, Mitchell Cancer Institute, University of South Alabama, Mobile, AL, USA

**Keywords:** DNA repair, alkylation damage, gastric cancer, DNA polymerase β, mismatch repair

## Abstract

DNA repair gene expression in a set of gastric cancers suggested an inverse association between the expression of the mismatch repair (MMR) gene MLH1 and that of the base excision repair (BER) gene DNA polymerase β (Polβ). To gain insight into possible crosstalk of these two repair pathways in cancer, we analysed human gastric adenocarcinoma AGS cells over-expressing Polβ or Polβ active site mutants, alone or in combination with MLH1 silencing. Next, we investigated the cellular response to the alkylating agent methyl methanesulfonate (MMS) and the purine analogue 6-thioguanine (6-TG), agents that induce lesions that are substrates for BER and/or MMR. AGS cells over-expressing Polβ were resistant to 6-TG to a similar extent as when MLH1 was inactivated while inhibition of O^6^-methylguanine-DNA methyltransferase (MGMT) was required to detect resistance to MMS. Upon either treatment, the association with MLH1 down-regulation further amplified the resistant phenotype. Moreover, AGS cells mutated in Polβ were hypersensitive to both 6-TG and MMS killing and their sensitivity was partially rescued by MLH1 silencing. We provide evidence that the critical lethal lesions in this new pathway are double strand breaks that are exacerbated when Polβ is defective and relieved when MLH1 is silenced. In conclusion, we provide evidence of crosstalk between MLH1 and Polβ that modulates the response to alkylation damage. These studies suggest that the Polβ/MLH1 status should be taken into consideration when designing chemotherapeutic approaches for gastric cancer.

## INTRODUCTION

More than thirty years ago the hypothesis of a mutator phenotype was postulated as a mechanistic basis for tumor generation [1]. This hypothesis predicts that a mutation in a gene that controls DNA replication fidelity and/or DNA repair capacity (so called “caretakers”) is a very early event in tumorigenesis, thus leading to an increased rate in the generation of mutations. Mutations in genes of the mismatch DNA repair (MMR) pathway are a typical example whereby the mutation (gene inactivation or dysfunction) triggers a chain of events that leads to a progressive accumulation of additional mutations. Subsequent mutations are predicted to increase the likelihood of alterations in additional genes involved in genome stability.

MMR reduces post-replicative errors in DNA, such as base-base mispairs and small insertion/deletion loops due to polymerase slippage. In this pathway many proteins combine to form repair complexes with different specificities. In humans, the heterodimer MutS α, formed by MSH2 and MSH6 proteins, binds single base mismatches or insertion/deletion loops [2], while the heterodimer MutS β, formed by MSH2 and MSH3, only binds to insertion/deletion loops [3]. The MUTL α heterodimer, formed by MLH1 and PMS2, binds to MutSα/β to initiate repair [4]. PCNA, EXO1 and DNA polymerase δ and ε catalyze the excision of the damaged base and the DNA resynthesis step [5]. Mutations or epigenetic silencing of MMR genes is associated with several human cancers of hereditary or sporadic origin including the colon and rectum, uterine endometrium, stomach, and ovaries [6]. For most sporadic cancers, inactivation of MLH1 is usually associated with methylation of the promoter, rather than mutation [7]. Deregulated expression of “caretakers” might also lead to increased genome instability. Over-expression of DNA replication/repair genes has been often described in tumor samples from a diverse range of tissues. Base excision repair (BER) is the main pathway for repair of damage generated by cellular metabolism and up-regulation of BER pathway proteins occurs in many types of solid tumors. BER is initiated by a DNA glycosylase that removes the base damage, followed by incision of the DNA backbone at the baseless (abasic) site by the major 5’ AP endonuclease APE1, and then restoration of the original template by DNA polymerase β (Polβ) re-synthesis and resealing by DNA ligase III/I. XRCC1 plays a general role as coordinator of BER via protein-protein interactions with DNA ligase III, PARP1, Polβ and PNK [8]. Polβ has been found to be over-expressed in approximately one third of cancer specimens in a screen involving different types of solid tumors [9]. Contrasting data about consequences of Polβ over-expression have been published. Some *in vitro* studies have shown that the over-expression of Polβ leads to apoptosis and to an increase in spontaneous mutation frequency and chromosomal aberrations [10, 11], while in other studies the over-expression of Polβ has been reported to not lead to cellular transformation [12] or to not alter cell growth or spontaneous DNA damage and genomic instability [13]. Upregulation of Polβ may occur by an increase in mRNA levels or by post-translation stabilization of the protein (by oxidative stress for instance) as shown in colon cancer cell lines resistant to oxaliplatin [14]. Such stabilization may be the result of alteration in the ubiquitylation of Polβ in cancers [15]. Polβ over-expression has been linked to increased spontaneous mutation frequency, resistance to anticancer drugs, aneuploidy and tumorigenesis [16-17]. Human tumors expressing Polβ variant proteins have been also described [18]. Some of these mutants synthesize DNA with a low fidelity or interfere with BER thus conferring a mutator cellular phenotype. BER seems to be vital for cancer cells as suggested by *in vitro* experiments where inhibition of APE1 and/or XRCC1 led to targeted cytotoxicity under acidic cellular environment conditions (i.e. low pH to mimick tumor microenvironment) [19].

Similarly to Polβ, APE1 is often over-expressed in tumors as compared to normal tissues [20-22]. Whether changes in the expression of BER components is a compensatory mechanism that follows the loss of another DNA repair pathway or it is due to an adaptive survival response in the acidic tumor microenvironment awaits to be addressed.

In this study, the analysis of the DNA repair gene expression profile of a set of human gastric cancer samples previously characterized for the microsatellite instability (MSI) status [23] revealed a high frequency of Polβ over-expression and an inverse correlation between MLH1 and Polβ expression. To mimic what we observed *in vivo*, gastric cancer cells with different MMR and BER genetic backgrounds were constructed and the impact of their DNA repair profile on the response to the monofunctional alkylating agent methyl methanesulfonate (MMS) and to the base analogue 6-thioguanine (6-TG) was analyzed. Simple methylating agents such as MMS form adducts on the N- and O-atoms of DNA bases, mostly N-alkylpurines that are a substrate for BER but also low levels of O^6^-methylguanine (O^6^-MeGua) that if left unrepaired by O^6^-MeGua-DNA-methyltransferase (MGMT) is processed by MMR. 6-TG incorporated into DNA is eventually methylated by S-adenosyl methionine (SAM) and then processed by MMR similarly to O^6^-MeGua. Alkylating agents and purine analogues such as 6-TG that kill cells via induction of DNA methylation of O^6^-guanines or S^6^-thyoguanines, respectively, are in use in gastric/colon cancer chemotherapy [24–25].

## RESULTS

### MLH1 and Polβ expression levels in gastric cancers are highly variable

**Figure 1:**
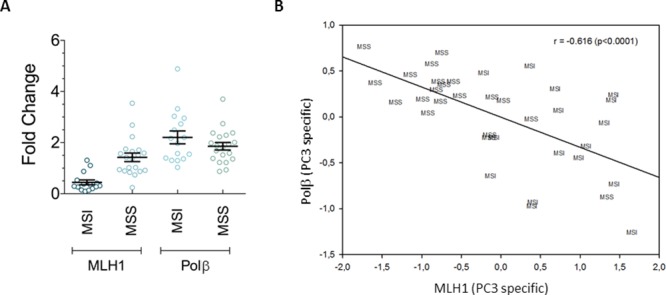
Variable expression levels of MLH1 and Polβ in gastric tumors **A.** Sporadic gastric cancers with MSI are characterized by MLH1 down-regulation and Polβ overexpression compared to MSS tumors. Horizontal black lines represent the mean. Error bars represent standard error **B.** Statistically significant correlation between the expression of MLH1 and Polβ along the PC3 pathway, identified by the covariance analysis.

The DNA repair gene expression profile of a set of gastric cancers with and without microsatellite instability (named MSI and MSS, respectively) has been previously characterised [23]. In this study we focused our analysis on the expression of two genes, MLH1 and Polβ, that are key players in MMR and BER, respectively. Figure 1 shows the heterogeneity of the expression levels of MLH1 and Polβ in our set of gastric tumors as determined by RT-PCR using low-density arrays. As expected from sporadic gastric cancers with MSI, MLH1 down-regulation (fold change<0.5 as compared to a pool of mRNA from normal gastric tissues) characterized 70% of these tumors as compared to only 5% of the MSS tumors (Figure 1A). In the case of Polβ, its over-expression (fold change>2) was detected more frequently among the MSS than the MSI tumors (50 and 30% of tumors, respectively) (Figure 1A). Moreover, principal component analysis of the overall DNA repair gene expression profile and covariance analysis (partial regression) on the principal components (see materials and methods, Supplementary Table S1) revealed that PC3, that accounted for 10% of the total variance, was able to identify a statistically significant inverse correlation between the expression levels of MLH1 and Polβ (Figure 1B). Overall these data indicate that the levels of expression of Polβ and MLH1 are very heterogeneous in MSI and MSS gastric cancers and suggest that there is a regulatory circuit, highlighted by PC3, where these two enzymes are inversely regulated.

### Effects of Polβ over-expression on the response to alkylating agents

#### Cytotoxicity

To gain insight into the relevance of the different expression levels of MLH1 and Polβ for the cytotoxic response of gastric cancers to chemotherapy, a series of recombinant gastric cancer cell lines were constructed where Polβ and MLH1 were differentially expressed and the response to different DNA damaging agents was investigated. Alkylating agents, namely MMS and N-methyl-nitrosourea (MNU), were selected because they induce DNA lesions that are a substrate for both BER (i.e. N-alkylpurines) and MMR (i.e. O^6^-MeGua). In order to study the role of the O^6^-MeGua adducts, O^6^-benzylguanine (O^6^-BzGua) was used as an efficient tool to inhibit MGMT and therefore to maximize the cytotoxic contribution of the O^6^-MeGua adducts. Cancer drugs based on the killing properties of O^6^-MeGua such as temozolomide are common in colon/gastric cancer chemotherapy particularly for those tumors with methylation of MGMT [24]. The cytotoxic response to the purine analogue 6-TG was used to specifically test the role of MMR that recognizes 6-meTG/T or C mismatches and, when defective, confers resistance to 6-TG. In addition, 6-TG is of use for advanced gastric carcinoma [25].

**Figure 2:**
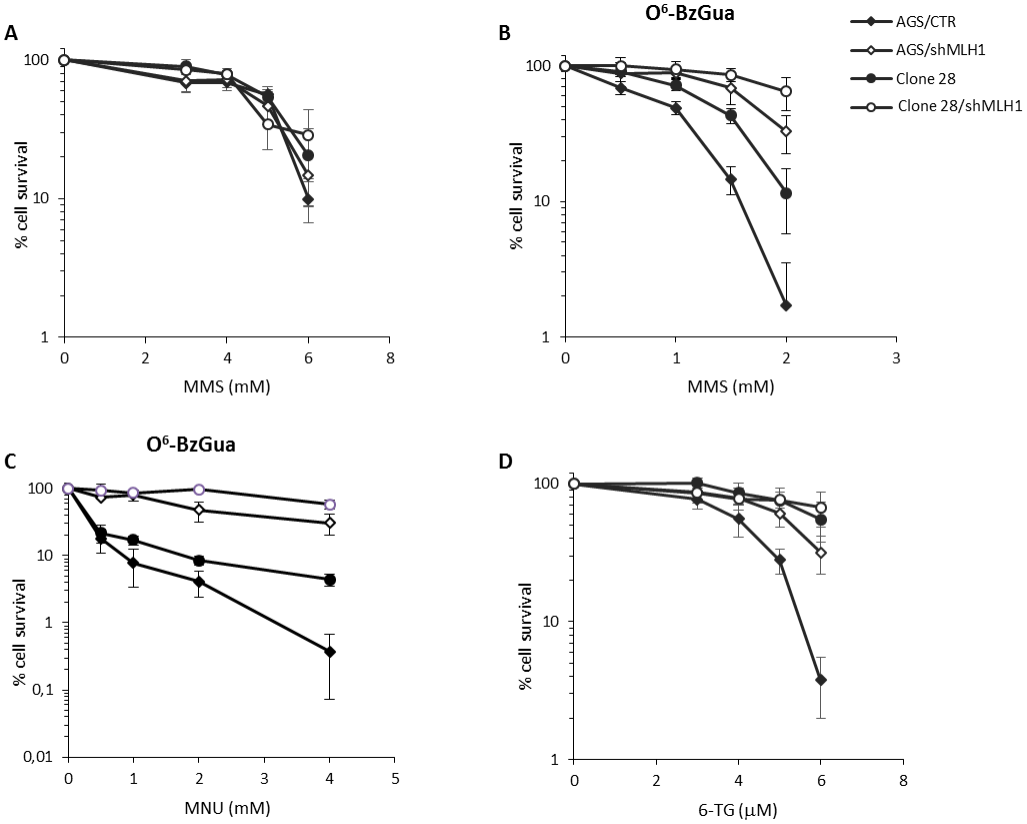
Survival response to alkylating agents of Polβ over-expressing gastric cancer cells with/without down-regulation of MLH1 **A.** Survival response after 30 minutes of treatment with 3, 4, 5, 6 mM MMS. **B.** Survival response after 30 minutes of treatment with 3, 4, 5, 6mM MMS in the presence of 25 μM O^6^-BzGua **C.** Survival response after 30 minutes of treatment with 0.5, 1, 2, 4 mM MNU in the presence of 25 μM O^6^-BzGua. **D.** Survival response after 7 days of exposure to 3, 4, 5, 6 μM 6-TG.

First, we analysed the biological effects of over-expression of Polβ associated with active or silenced MLH1 with regard to the response to MMS and 6-TG. Gastric cancer clonal cell lines expressing different levels of Polβ were isolated following transfection of the AGS cell line with a mammalian expression vector containing Polβ cDNA. Clones obtained were highly variable for Polβ expression as detected by RT-PCR (data not shown). Among them, we selected a clone with an 8-fold increase in the level of Polβ mRNA (clone 28) over control (AGS/CTR) together with a pool of transfected AGS clones with an average 2-fold increase in Polβ expression (indicated as Pool) that resembles what was observed in our set of gastric cancers (Supplementary Figure S1A). Next, the cytotoxic response to the selected agents was measured in colony survival assays. In clone 28, over-expression of Polβ alone or in combination with MLH1 down-regulation (by shRNA) did not affect the response to the lethal effect of MMS (Figure 2A). However, when the exposure to MMS was performed in the presence of the MGMT inhibitor O^6^-BzGua, clone 28 showed a significant recovery of survival as compared to control cells expressing physiological levels of Polβ (Figure 2B). Under these experimental conditions, when MLH1 was down-regulated by shRNA, an increased resistance to MMS was observed in wild-type cells, as expected. In addition, the resistance of Polβ over-expressing cells increased further (p<0.05, Student’s t-test). Since these data suggested that Polβ over-expression is able to modulate the cytotoxic effects of O^6^-MeGua, to strengthen this association clone 28 cells were exposed to the SN1 alkylating agent MNU that induces relatively higher levels of alkylation at O^6^-MeGua than MMS. In the presence of O^6^-BzGua, the resistance of clone 28 to MNU as well as the increased resistance due to MLH1 down-regulation was confirmed (Figure 2C). Consistently with what we observed upon exposure to alkylating agents under MGMT saturation, over-expression of Polβ was able to significantly increase resistance to 6-TG (p<0.05, Student’s t-test) (Figure 2D). When MLH1 was down-regulated, we observed the expected increased resistance to 6-TG of wild-type cells. In addition, the resistance of AGS cells expressing Polβ (clone 28), suggested an increase in resistance associated with Polβ over-expression. Next we evaluated the pooled clones that show a 2-fold increase in Polβ expression. These cells showed intermediate resistance following either MMS (Supplementary Figure S1B) or 6-TG (Supplementary Figure S1C) exposure indicating that the resistant phenotype is associated with Polβ expression levels.

The enhanced resistance of clone 28 cells to alkylating agents (when MGMT is inhibited) and to 6-TG as well as the potentiation of this effect when MLH1 is down-regulated suggest that Polβ over-expression may counteract the lethal effects of O^6^-methyl guanine-related lesions.

#### Double strand break repair

**Figure 3:**
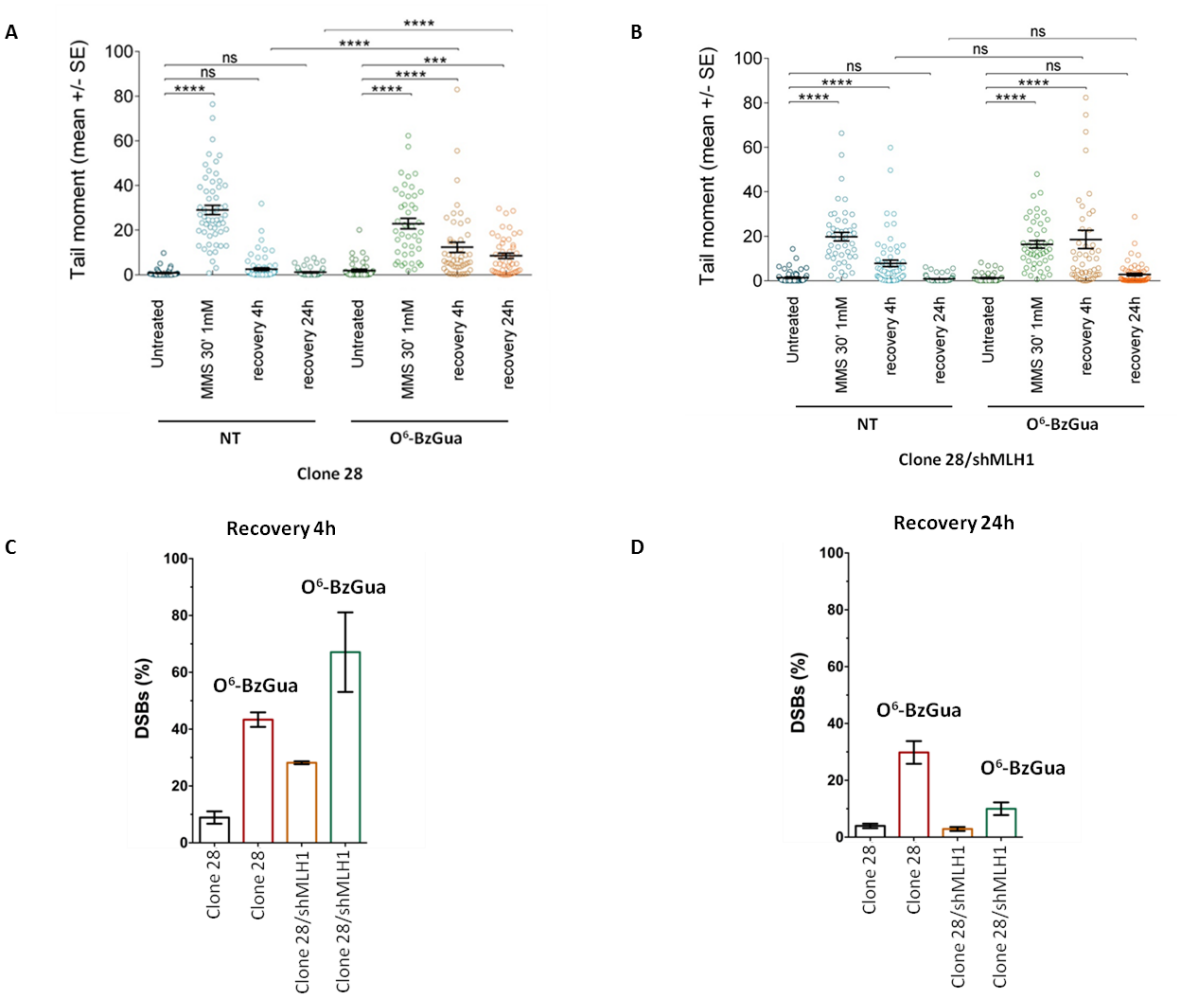
Analysis of DSB formation in Polβ over-expressing gastric cancer cells with/without down-regulation of MLH1 evaluated by neutral Comet assay Cells were treated with 1 mM MMS for 30 min, in the presence or absence (NT) of O^6^-BzGua. Dot plot shows tail moment per cell. Horizontal black lines represent the mean. Error bars represent standard error (ns, not significant; ****, P < 0.0001; Kruskal-Wallis test multicomparison Anova). **A.** DSB repair in clone 28 at 4h and 24h recovery time **B.** DSB repair in clone 28/shMLH1 at 4h and 24h recovery time **C.** Levels of persisting DSB at 4h post-treatment normalized for the level detected immediately after treatment. **D.** Levels of persisting DSB at 24h post-treatment normalized for the level detected immediately after treatment.

To gain insight into the mechanisms underlying the resistant phenotype of Polβ over-expressing cells, a fine characterization of MMS-induced DNA double strand breaks (DSBs) and their repair was conducted by neutral comet, in the presence or absence of O^6^-BzGua. As shown in Figure 3A, Clone 28 repairs DSBs induced by MMS very efficiently following a 4h recovery period and no breaks are detected at 24h. When the treatment is performed in the presence of O^6^-BzGua significantly increased DSB are detected at both 4 and 24h recovery. When MLH1 is down-regulated (Figure 3B) persisting breaks are observed at 4h recovery and these are further increased in the presence of O^6^-BzGua. To facilitate comparisons, the levels of persisting DSBs at 4 and 24h post-treatment times (normalized for the level detected immediately after treatment) for all experimental conditions are presented in Figure 3C and 3D, respectively. DSBs detected at 4h recovery (Figure 3C) are significantly increased in the presence of O^6^-BzGua as well as when MLH1 is silenced and MGMT is inactivated by O^6^-BzGua. Conversely, DSBs detected at 24h recovery times (Figure 3D) in the presence of O^6^-BzGua are drastically decreased when MLH1 is down-regulated thus indicating that they are MMR dependent. These breaks are expected to arise from MMR events at O^6^-MeGua/T mismatches (see also Figure 6).

These findings suggest that Polβ over-expression leads to the transitory formation of DSBs (at 4h post-treatment time) that involves O^6^-MeGua adducts and are mostly independent of MLH1. The profile of DSBs at 24h recovery seems to be unaffected by Polβ over-expression but fully dependent on MLH1 expression (Figure 3B).

### Effects of Pol β inactivation on the response to alkylating agents

#### Cytotoxicity

**Figure 4:**
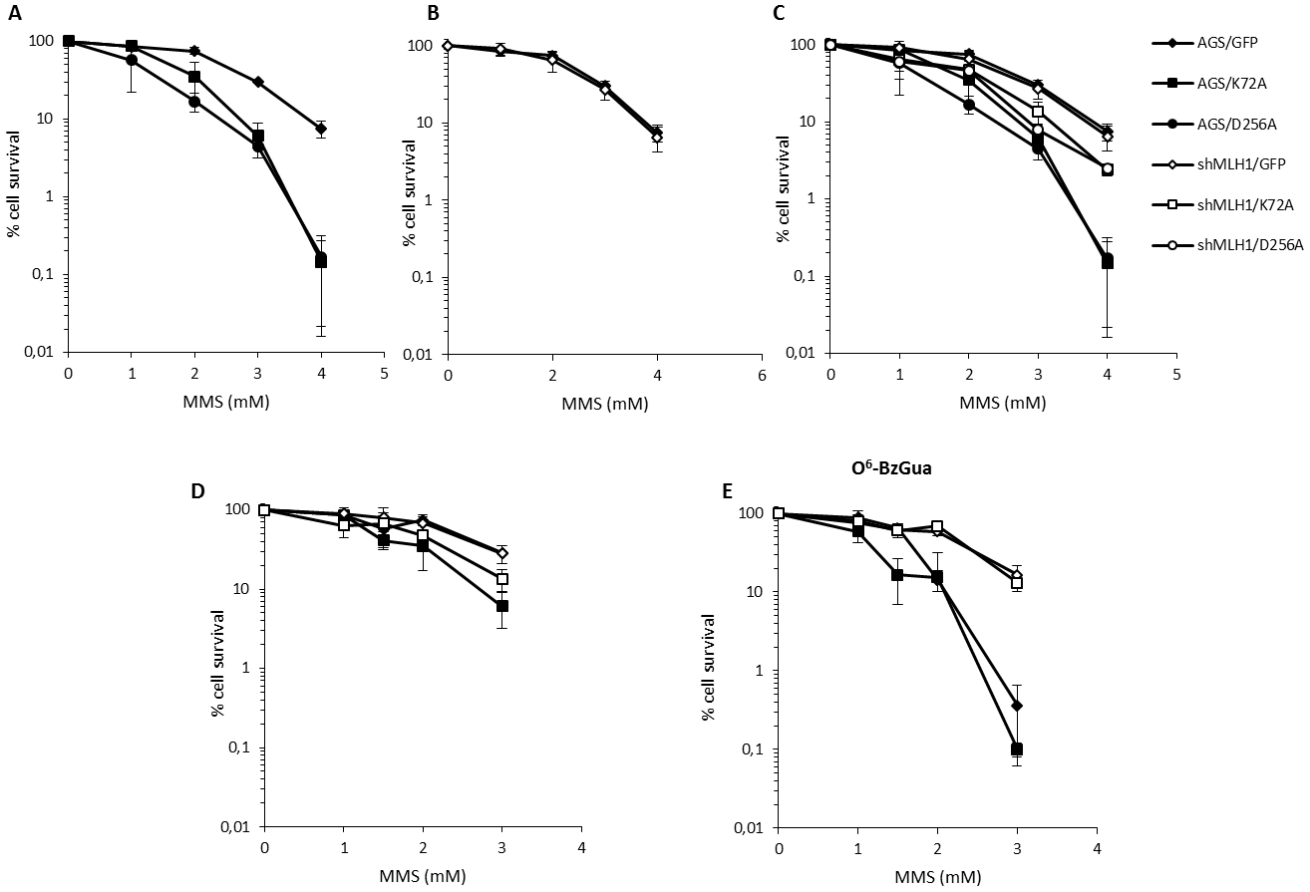
Survival response to MMS of Polβ mutant over-expressing gastric cancer cells with/without down-regulation of MLH1 **A.** Survival response of AGS/GFP, AGS/K72A and AGS/D256A after 30 min treatment with 1, 2, 3, 4 mM MMS. **B.** Survival response of AGS/GFP and shMLH1/GFP after 30 min treatment with 1, 2, 3, 4 mM MMS. **C.** Survival response of AGS/GFP, AGS/K72A, AGS/D256A, shMLH1/GFP, shMLH1/K72A and shMLH1/D256A after 30 min treatment with 1, 2, 3, 4 mM MMS. **D.** Survival response of AGS/GFP, AGS/K72A, shMLH1/GFP and shMLH1/K72A after 30 min treatment with 1, 1.5, 2, 3 mM MMS. **E.** Survival response of AGS/GFP, AGS/K72A, shMLH1/GFP and shMLH1/K72A after 30 min treatment with 1, 1.5, 2, 3 mM MMS in the presence of 25 μM O^6^-BzGua.

To better analyze the potential crosstalk between MMR and BER, recombinant cell lines over-expressing Polβ active site mutants were obtained by lentiviral-mediated transduction of the AGS control and AGS/MLH1-KD cells. The mutants are within the dRP lyase domain (K72A) or in the DNA polymerase domain (D256A). Colony survival assays were performed by using AGS cells of all genotypes: AGS/GFP vector (AGS/GFP), AGS over-expressing Polβ mutants (AGS/K72A, AGS/D256A), AGS with down-regulation of MLH1 (shMLH1) and AGS with down-regulation of MLH1 and over-expression of Polβ mutants (shMLH1/K72A, shMLH1/D256A). As previously shown in rodent cells [26], inactivating mutations of the dRP lyase domain of Polβ caused a significant (p< 0.05, Student’s t-test) increase in sensitivity to MMS, compared to control AGS/GFP cells. Interestingly, mutation in the polymerase domain of Polβ conferred also a hypersensitive phenotype to AGS cells (p< 0.05, Student’s t-test) (Figure 4A). The silencing of MLH1 did not affect the sensitivity to MMS (Figure 4B) but, very surprisingly, it was able to alleviate sensitivity of AGS/K72A and AGS/D256A to MMS (p< 0.05, Student’s t-test) (Figure 4C). When this experiment was performed in the presence of O^6^-BzGua, besides a general increased toxicity for all strains, the only relevant effect observed was the increased sensitivity of the control AGS/GFP cells that reached levels of cytotoxicity similar to those of the dRP Polβ mutant over-expressing cell line (Figure 4E).

**Figure 5:**
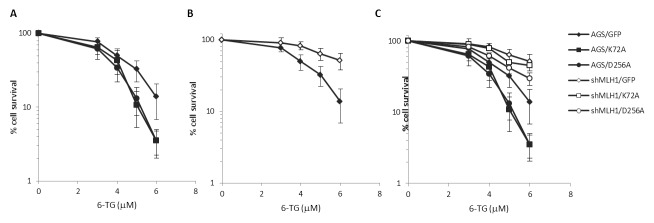
Survival response to 6-TG of Polβ mutant over-expressing gastric cancer cells with/without down-regulation of MLH1 **A.** Survival response of AGS/GFP, AGS/K72A and AGS/D256A after 7 days of exposure to 3, 4, 5, 6 μM 6-TG. **B.** Survival response of AGS/GFP and shMLH1/GFP after 7 days of exposure to 3, 4, 5, 6 μM 6-TG. **C.** Survival response of AGS/GFP, AGS/K72A, AGS/D256A, shMLH1/GFP, shMLH1/K72A and shMLH1/D256A after 7 days of exposure to 3, 4, 5, 6 μM 6-TG.

To further analyze this phenomenon, colony survival assays were performed next with 6-TG. Similar to what we observed with MMS treatment, both Polβ mutations caused a modest but statistically significant (p< 0.05, Student’s t-test) increase in sensitivity to 6-TG and the silencing of MLH1 partially rescued survival (p< 0.05, Student’s t-test) (Figures 5A and 5C). As expected from a defect in MMR, AGS/shMLH1 cells were more resistant than AGS/GFP cells to 6-TG (Figure 5B).

In the absence of Polβ, unrepaired BER intermediates (i.e. single strand breaks) arising from MMS treatment are expected to give rise to lethal events. Interestingly, here we show that a fraction of these lethal events is mediated by MLH1 since its silencing partially relieves the toxic effects of MMS. In the absence of the toxic effect of N-alkylpurines, the treatment with 6-TG allows us to identify a similarly protective effect mediated by MLH1 down-regulation. This phenomenon is expected to involve O^6^-MeGua lesions.

#### Double strand break repair

**Figure 6:**
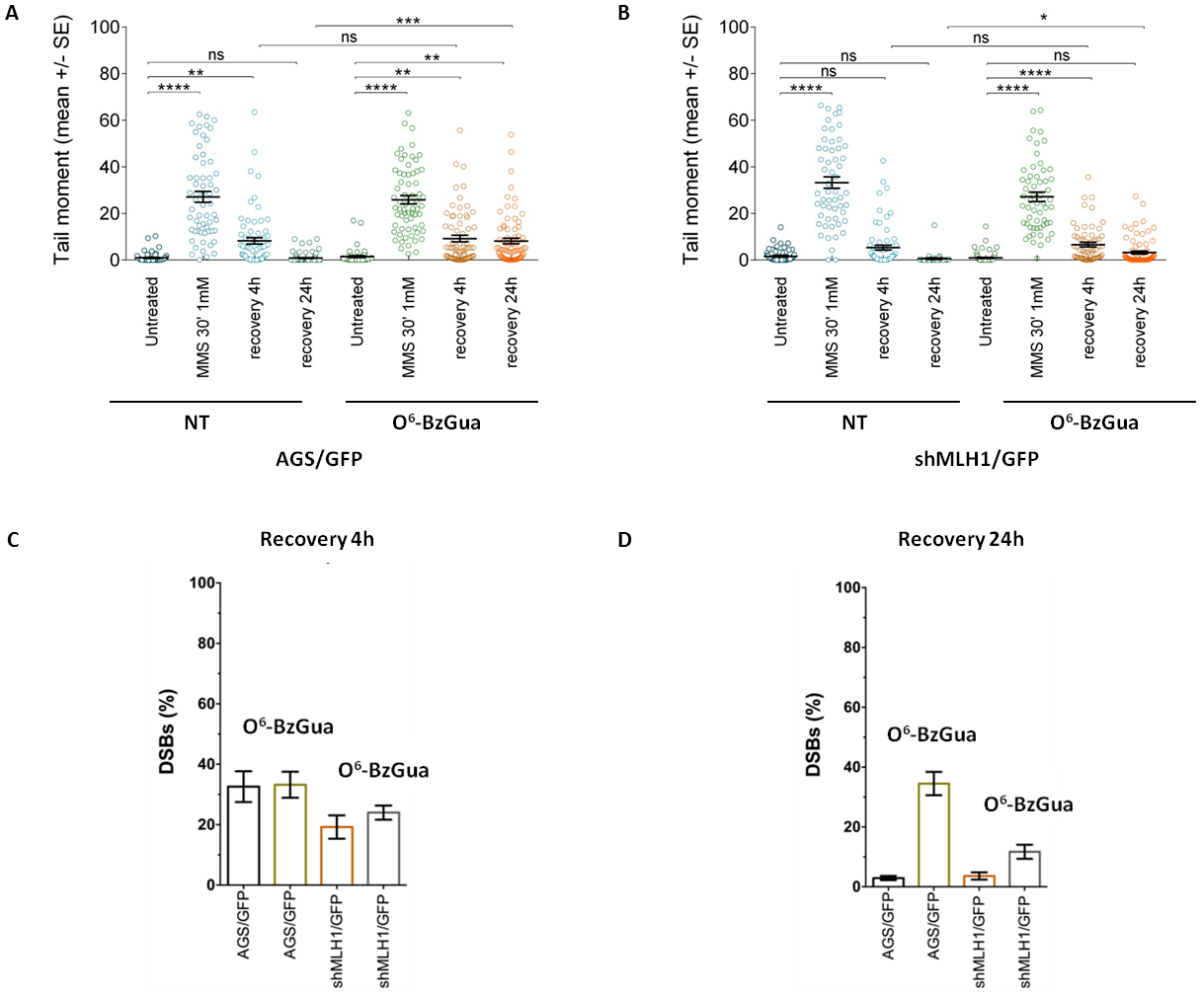
Analysis of DSB formation in AGS gastric cancer cells with/without down-regulation of MLH1 evaluated by neutral Comet assay Cells were treated with 1 mM MMS for 30 min, in the presence or absence (NT) of O6-BzGua. Dot plot shows tail moment per cell. Horizontal black lines represent the mean. Error bars represent standard error (ns, not significant; ****, P < 0.0001; Kruskal-Wallis test multicomparison Anova. **A.** DSB repair in AGS/GFP at 4h and 24h recovery time **B.** DSB repair in shMLH1/GFP at 4h and 24h recovery time **C.** Levels of persisting DSB at 4h post-treatment normalized for the level detected immediately after treatment. **D.** Levels of persisting DSB at 24h post-treatment normalized for the level detected immediately after treatment.

The induction and repair of MMS-induced DSBs was measured by neutral comet in all Polβ mutant over-expressing cell lines, in the presence or absence of O^6^-BzGua. As shown in Figure 6A, in normal cells almost 70% of these breaks are repaired after 4h recovery and, consistent with their generation from N-alkylpurines, their levels are unaffected by O^6^-BzGua-mediated MGMT inhibition (Figure 6A) or by MLH1 down-regulation (Figure 6B). At the 24h recovery time, DSBs are detected in AGS cells only in the presence of O^6^-BzGua (Figure 6A) but not when MLH1 is down-regulated (Figure 6B) since they require active MMR and inhibition of MGMT (by O^6^-BzGua). The levels of persistent DSBs at 4 and 24h post-treatment times (normalized for the level detected immediately after treatment) for all experimental conditions are presented in Figure 6C and 6D, respectively. These data very clearly show that BER events (that are unaffected by O^6^-BzGua and MLH1 down-regulation) are detected at 4h recovery while MMR events at O^6^MeGua/T mismatches generated by replication of persistent O^6^-MeGua adducts (not repaired by MGMT) are detected at 24h recovery. These DSB require two rounds of replication to be produced [27] thus explaining their late detection at 24h post-treatment time.

**Figure 7:**
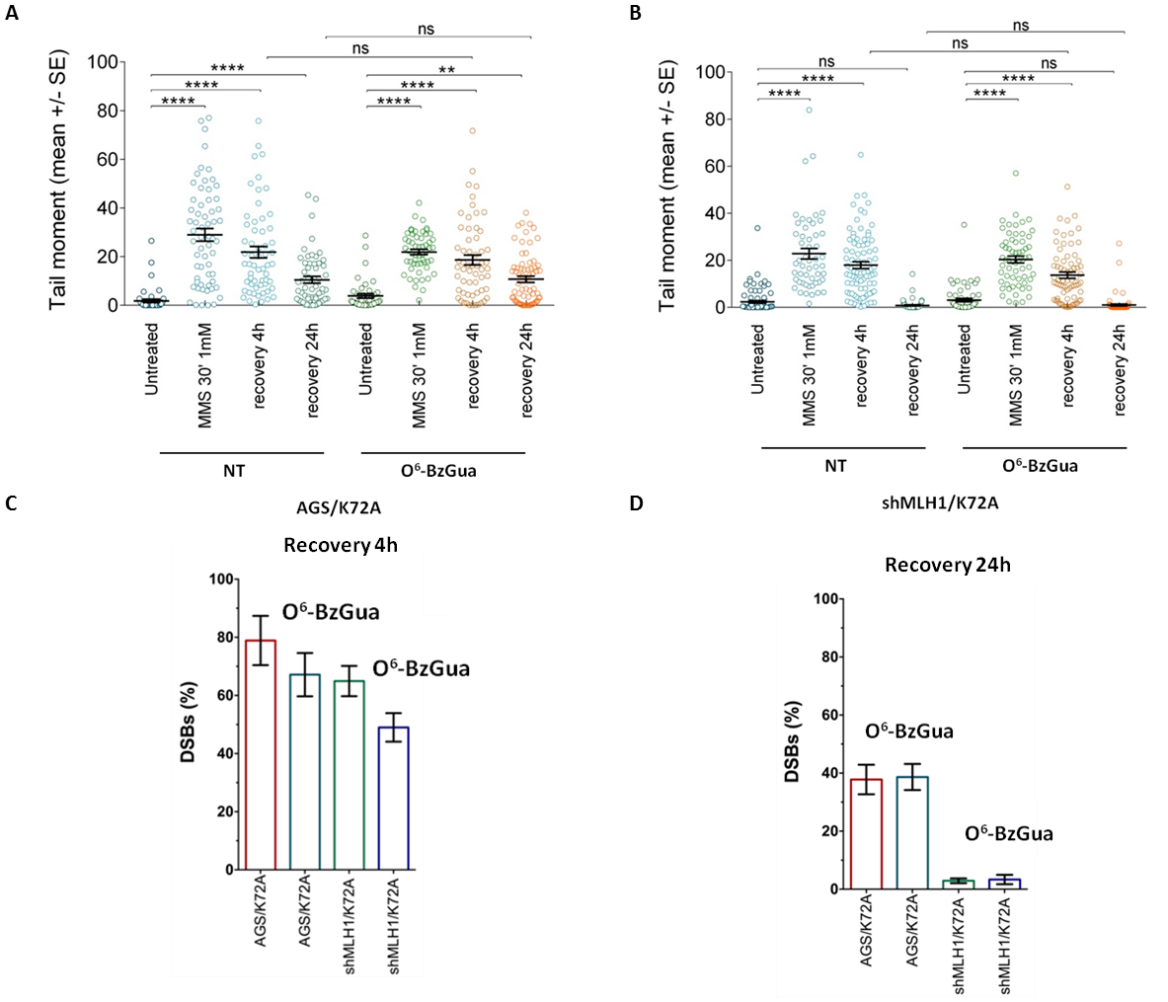
Analysis of DSB formation in Polβ mutant over-expressing gastric cancer cells with/without inactivation of MLH1 evaluated by neutral Comet assay Cells were treated with 1 mM MMS for 30 min, in the presence or absence (NT) of O^6^-BzGua. Dot plot shows tail moment per cell. Horizontal black lines represent the mean. Error bars represent standard error (ns, not significant; ****, P < 0.0001; Kruskal-Wallis test multicomparison Anova). **A.** DSB repair in AGS/K72A at 4h and 24h recovery time **B.** DSB repair in shMLH1/K72A at 4h and 24h recovery time **C.** Levels of persisting DSB at 4h post-treatment normalized for the level detected immediately after treatment. **D.** Levels of persisting DSB at 24h post-treatment normalized for the level detected immediately after treatment.

Figure 7 shows the kinetics of DSB repair in Polβ mutant over-expressing cells. As shown in Figure 7A, defective Polβ leads to a significant accumulation of DSBs at 4h recovery time and their level is not affected by O^6^-BzGua-mediated MGMT inhibition. At 24h recovery, DSBs are still detectable when no breaks are observed in normal cells (compare with Figure 6A) and they are unaffected by O^6^-BzGua. When MLH1 is inactivated (Figure 7B), no significant change in DSB levels are observed at 4h recovery also in the presence of O^6^-BzGua while no breaks are detected at 24h recovery either in the absence or in the presence of O^6^-BzGua. The inactivation of Polβ that leads to 50-80% unrepaired DSBs (that are unaffected by O^6^-BzGua and MLH1 down-regulation) at 4h recovery (Figure 7C) and to a significant persistence of DSBs at 24h recovery (40% remaining DSB versus no breaks in normal cells, Figure 7D). The accumulation or formation of these DSBs is however counterbalanced by down-regulation of MLH1 (at 24h recovery) that leads to a drastic reduction of DSBs both in the presence and in the absence of O^6^-BzGua. This effect is in-line with the attenuated MMS-induced lethality observed in Polβ mutant cells when depleted of MLH1 (Figure 4C). No effect of the different pattern of DSB repair on cell cycle were detected (supplementary Figure S2).

Overall these findings indicate that upon MMS exposure, DSBs originate from either BER or MMR events with different kinetics, at early times (4h recovery) in the case of BER-derived DSBs and at late times (24h recovery) in the case of MMR-derived DSBs. Interestingly, when Polβ is defective, a sub-pathway emerges where DSBs arise from overlapped MMR and BER events.

## DISCUSSION

In this study we addressed the question of whether the relative expression level of Polβ and MLH1, that show a highly heterogeneous expression profile in our set of gastric cancers, might impact on the cellular response to different types of DNA damage commonly induced by chemotherapeutic drugs.

Gastric cancer cells over-expressing Polβ (common trait in stomach tumors) present increased resistance to 6-TG and also to MMS upon inhibition of MGMT by O^6^-BzGua (that is in clinical trials). Under these conditions, the deregulation of Polβ leads to very efficient repair of DSBs but also to the transitory formation of DSBs (at 4h recovery) that persist also when MLH1 is silenced (Figure 3) thus suggesting that these breaks are created at O^6^-MeGua lesions but independently of MMR. It has been proposed that Polβ, when over-expressed, acts as a genetic instability enhancer by interference in replicative DNA synthesis [28]. Structural studies suggest that Polβ may replicate, albeit slowly, across O^6^-MeGua lesions [29]. We can hypothesize that Polβ interferes with replication opposite O^6^Me Gua lesions creating ssDNA gaps and thus increasing the likelihood of DSB. The pro-mutagenic replication across O^6^-MeGua lesions by excessive Polβ would result in gain of survival (as suggested by our data) although at the expense of replication fidelity. In-line with our data, Luo et al [13] showed that, upon MMS exposure, an excessive amount of Polβ promotes an increase in *hprt* mutation frequency, presumably through an error-prone repair response, although it enhances overall BER capacity for induced DNA damage. The resistance to 6-TG and MMS of Polβ over-expressing cells is further increased by the concomitant down-regulation of MLH1, indicating that canonical MMR and the resistance mediated by Polβ over-expression are independent pathways. On the basis of our data, we may conclude that over-expression of Polβ associated with silencing of MLH1 provides a growth advantage to gastric cancer cells in the presence of damage to the detriment of genetic integrity.

**Figure 8:**
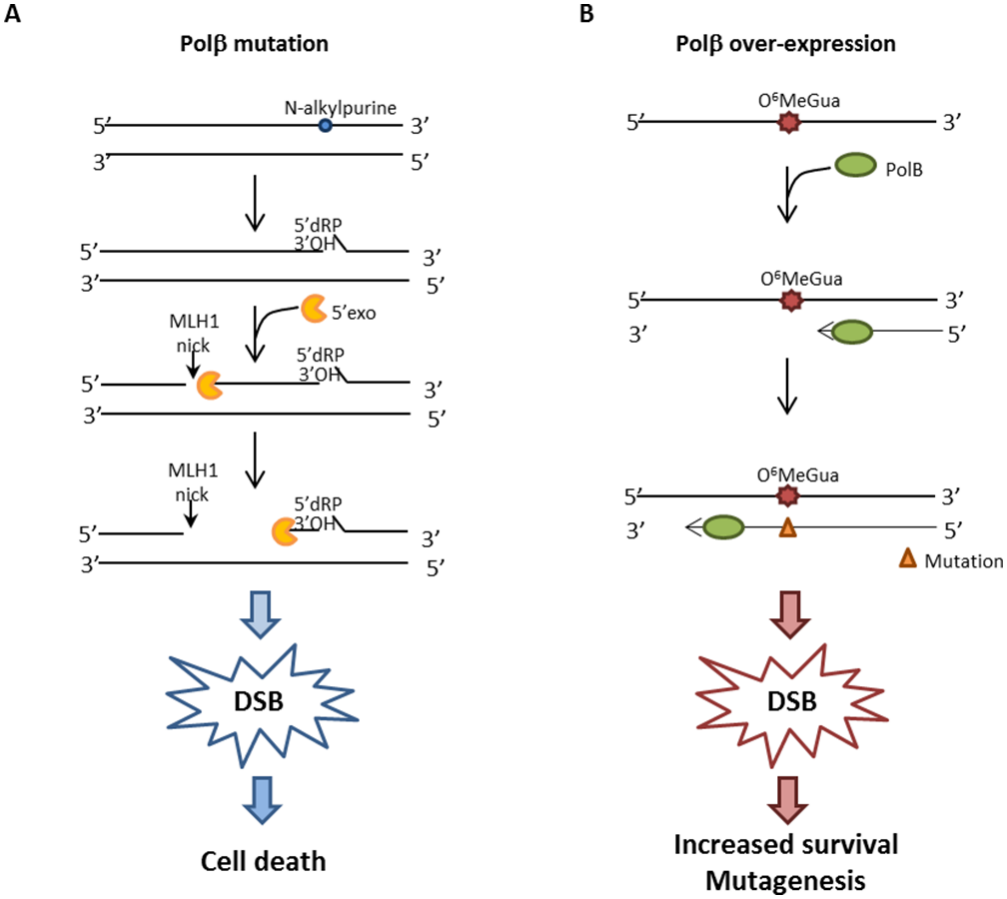
Hypothetical model for DSB formation **A.** When Polβ is mutated, BER is impaired and MMR initiates the processing of unrepaired BER intermediates. MLH1 introduces a nick 5’position to the lesion and Exo1 then subsequently produces a single-stranded DNA gap which might be inefficiently repaired outside of S-phase. **B.** When Polβ is over-expressed, it improperly participates in DNA synthesis opposite O^6^-MeGua lesions, leading to a mutation. Reduced Polβ processivity causes a delay in DNA synthesis, thus leading to the formation of transitory DSBs.

When Polβ is inactive, either because it is mutated in the DNA polymerase or in the dRP lyase active site, we show that gastric cancer cells are significantly sensitized to killing by MMS. It has been shown that N-methylpurine DNA glycosylase (MPG) and Polβ expression predict the sensitivity to the alkylating cancer drug temozolomide [30] and that Polβ-dependent 5’dRP lyase activity is the rate-limiting step in BER in breast cancer cells. Our data indicate that gastric cancer cells that are Polβ defective are extremely sensitive to the lethal effect of simple model alkylating agents such as MMS likely because of the production of cytototoxic 1-nt gaps and 5’dRP residues during N-alkylpurine processing. These BER repair intermediates are expected to increase the likelihood of DSBs when replication occurs [31] and this is indeed the case as shown by the high level of persistant DSBs when Polβ is defective at both early and late times of recovery (Figure 7). Interestingly, when MLH1 is silenced and Polβ is mutated, a resistant phenotype due to the MMR defect emerges partially alleviating the sensitivity to MMS and this is associated with a decrease in the persisting DSB at 24h recovery when cells are depleted of MLH1 (Figure 7). This phenomenon is not affected by O^6^-BzGua indicating that it does not involve O^6^-MeGua lesions but only BER lesions. These data are compatible with a model (Figure 8) where, when the BER pathway is not coordinated (because of defective Polβ), MMR has access to BER intermediates creating ssDNA gaps that would then lead to DSBs. This is consistent with their reduction when depleting factors involved in MMR (i.e. MLH1). In the absence of MLH1 homologous recombination is expected to take place thus alleviating the DSB-driven lethality [31–32]. Increased survival following MLH1 down-regulation is also observed when Polβ mutant over-expressing cells are exposed to 6-TG suggesting that also O^6^-MeGua lesions may also be involved in this new “tolerance” pathway.

Jiricny’s laboratory has provided clear evidence [33] that MMR may recognize lesions, including alkylation damage, outside of S-phase. This pathway is largely independent of DNA replication, lacks strand directionality, induces PCNA monoubiquitylation and promotes recruitment of translesion synthesis (TLS) DNA polymerases. Nicks introduced in DNA by MUTLα would then result in the generation of long ssDNA by EXO1 and the unrepaired gaps would be converted into DSBs. We have previously shown that alkylation damage induces PCNA monoubiquitylation and involves the recruitment of the TLS polymerase Polκ that modulates the cytotoxic effects of O^6^-MeGua [34]. More recently, BER hijacking by MMR has been invoked as a mechanism to account for increased mutations following processing of BER lesions and depleting factors involved in MMR resulted in a reduction of the mutational load [35–36].

On the basis of the present findings, we propose that a small portion of alkylation-induced Polβ-unattended repair intermediates (so-called BER failure) can be recognized by MMR and will give rise to lethal DSBs.

In conclusion the findings of this study provide evidence of crosstalk between Polβ and MLH1. Polβ, when over-expressed, is an important alkylating drug resistance factor that acts independently of a functional MMR pathway. However, if MMR is also defective, this will lead invariably to increased alkylation resistance. Moreover, we suggest that MMR may operate at Polβ-unattended repair intermediates leading to DSBs and likely contributing to genetic instability in cancer. From these studies, we suggest that both MMR and BER status should be investigated to tailor therapy in the treatment of gastric cancer.

## MATERIALS AND METHODS

### Principal component analysis of the DNA repair gene expression profile

Human gastric tissue collection and their DNA repair gene expression profiling has been previously described [23]. Briefly, 36 tumor samples with and without microsatellite instability (MSI) and matched normal mucosa were analysed by real-time reverse-transcription PCR (RT-PCR) using pre-designed low density arrays (Applied Biosystems) for gene expression of selected DNA repair genes (APEX1, MLH1, BRCA1, BRCA2, ERCC1, FEN1, LIG1, LIG3, LIG4, MBD4, MPG, MRE11A, MSH2, MSH3, MSH6, OGG1, PMS2, POLΒ, RAD51, SMUG1, UNG, XPC, XRCC1). Gene expression data were analysed by principal component analysis (PCA). The difference in gene expression between tumor and normal gastric tissues was evaluated using as the calibrator sample a pool of mRNA from normal gastric tissues.

Low-density gene expression data were submitted to PCA by using as rows (statistical units) the samples and as columns (variables) the different gene expression levels. PCA allows for the projection of an initially N-dimensional space (with N being the number of variables) into a lower dimensional one relying on the ‘between variables mutual correlation’. The axes of this derived space are called principal components and are each other independent by construction [37].

A three-component solution (PC1-PC3) accounted for 65% of the total variance (PC1 = 41%, PC2 = 13%, PC3 = 9%) with a clear separation of the three signal components from the noise floor [38]. Inferential and descriptive statistics highlighted a marked significance of PC3 as for discrimination of gastric cancer with MSI or without MSI (microsatellite stable, MSS) (Student’s t-test (PC3) = 3.36, p < 0.002), while both PC1 and PC2 did not show any statistically significant difference between the two groups. The plane spanned by PC1 and PC3 loadings allowed the visualization of the discrimination between MSS and MSI tumors (Figure 1B). Component loading matrix [39] (supplementary, Table S1) shows a peculiar pattern for PC3 with high values (positive correlation) of Pol β associated with low values of MLH1 (negative correlation).

Given the specific biological interest of PC3, a covariance analysis was performed, defining the contribution of PC1 and PC2 on the MLH1-Polβ relationship, keeping alive only the PC3 contribution. This was achieved by operating a correlation on the transformed variables:

MLH1(pc3specific) = MLH1 – MLH1 est (PC1, PC2)

Polβ (PC3 specific) = Polβ – Polβ est (PC1, PC2)

(est stands for estimated)

Where MLH1 and Polβ are the raw variables, while MLH1 est (PC1, PC2) and Polβ est (PC1, PC2) are the least squares estimation of MLH1 and Polβ respectively, by means of PC1 and PC2 scores (namely MLH1 est = 19.31 +0.485 (PC1) + 0.644 (PC2), Pearson r = 0.65 (p<0.0001); Polβ est = 18.60 + 0.326(PC1) + 0.092(PC2), Pearson r = 0.56 (p<0.002]. The above operation allows to us single out the ‘pure’ PC3 effect on the MLH1-Polβ relationship.

### Construction of recombinant cell lines

#### Construction of Polβ over-expressing AGS cell lines

Wild type Polβ cDNA molecule from AGS cells (purchased from ATCC) were cloned into pcDNA4/HisMax (Invitrogen) expression vector, by using one-step cloning strategy (“TOPO Cloning”, Invitrogen) and then transfected into AGS cell line. The resulting vector will express human Polβ with an N-terminal tag consisting of 6xHis, the Xpress epitope and the EK recognition site, adding 33 amino acids (∼4 kDa) to the N-terminus. Empty vectors were used as control. Transfection was performed by using lipofectamine 2000 (Invitrogen) according to the manufacturer’s protocol and 48h from transfection zeocin (CAS # 181494-14-4, Invitrogen) was added at 125 μg/ μl final concentration. Cells were then seeded at low density to isolate clones with different levels of Pol β over-expression. The over-expression of Polβ in the selected clone, clone 28, was measured by real-time PCR (Supplementary, Figure S1A) and western blotting (Supplementary, Figure S3).

#### Construction of MLH1-silenced AGS cell lines

MLH1 was silenced in AGS cell lines containing pcDNA4/HisMax empty vector (AGS/CTR) and clone 28 by using the MISSION shRNA Library from Sigma-Aldrich, according to the manufacturer’s instructions. The silencing of MLH1 was monitored by qRT-PCR.

#### Construction of Polβ mutant over-expressing AGS cell lines

AGS/CTR or MLH1-silenced cells were modified by lentiviral-mediated expression of EGFP, wild-type Polβ or two Polβ active site mutants: D256A, defective in DNA polymerase activity, or K72A, defective in dRP lyase activity. Cell lines were developed by lentiviral transduction, stable integration and selection. Lentiviral particles were generated by co-transfection of 4 plasmids (Control plasmid, pLVX-EGFP-IRES-puro) or the Polβ expression plasmids pLVX-Flag-Pol β(WT)-IRES-puro, pLVX-Flag-Pol β(D256A)-IRES-puro or pLVX-Flag-Pol β(K72A)-IRES-puro together with pMD2.g(VSVG), pVSV-REV and pMDLg/pRRE] into 293-FT cells using FuGene 6 Transfection reagent, essentially as we have described [15]. Forty-eight hours after transfection, lentivirus-containing supernatant was collected and passed through 0.45 μM filters to isolate the viral particles. Lentiviral transduction was performed as follows: Cells (6 × 10^4^) were seeded into a 6-well plate 24 hours before transduction. Lentiviral particles were mixed with polybrene (2μg/ml) and then added to the cells, incubating at 32°C overnight. Cells were then cultured for 72 hours at 37°C and were then selected by culturing in selection medium for 1-2 week(s). 20-30μg nuclear extracts were analyzed by immunoblotting to determine the expression of the desired proteins. All cells were cultured at 5% CO_2_ and 37°C.

The control cell line (AGS/GFP) of this set of recombinant cells differs from the control cell line of clone 28 (AGS/CTR) for the presence of one more empty vector (pLVX-EGFP-IRES-puro).

All recombinant cell lines were checked for MGMT levels by western blotting (Novus Biologicals). Similar levels of MGMT were measured in all cell lines (Supplementary Figure S4)

### Colony survival assay

AGS recombinant cell lines were seeded at low density, depending on cloning efficiency of each cell line, onto 60-mm dishes in triplicate for each dose tested. Cells were treated with MMS (Sigma-Aldrich) at the indicated concentrations for 30 minutes. Then, the medium was replaced and cells were grown for 7 days, fixed with 100% ethanol and stained with Giemsa. In the case of 6-TG, the cells were incubated with the drug for 7 days. Then cells were processed as described above. Colonies containing 50 or more cells were counted. At least three independent experiments were performed for each agent.

### Measurement of double-strand breaks by the neutral Comet assay

The occurrence of DNA double-strand breaks was evaluated by neutral Comet assay as previously described [40]. Cell DNA was stained with GelRed (Biotium) and examined at 40× magnification with an Olympus fluorescence microscope. Slides were analyzed by a computerized image analysis system (Comet IV, Perceptive UK). To assess the amount of DNA damage, computer-generated tail moment values (tail length × fraction of total DNA in the tail) were used. A minimum of 300 cells was analyzed for each experimental point. Apoptotic cells (smaller comet head and extremely larger comet tail) were excluded from the analysis to avoid artificial enhancement of the tail moment. At least two independent experiments were performed.

### Cell cycle analysis

To evaluate the MMS-induced perturbations in cell cycle progression, cells were grown for 24h in culture medium, then pulse-labeled with 30 μM BrdU (Life Technologies Corporation) for 30 min. After extensive washing, cells were collected and fixed in 90% cold ethanol on ice for at least 1h. Cells were processed for flow cytometry as follows: after fixation, cells were exposed to acid denaturation (2 N HCl), neutralization buffer (0.1 M sodium tetraborate) and blocking solution (10% NGS/PBS). After that, cells were incubated with an anti-BrdU fluorescently-labeled antibody (eFluor® 450, eBioscience). Samples were resuspendend in 20 μg/ml propidium iodide (Sigma-Aldrich, St. Louis, MO, USA). Cytofluorimetric acquisition was done on a BD FACScalibur using CellQuest software (BD Biosciences, San Jose, CA, USA), and analyses were performed using FlowJo software v. 7.6.5 (Tree Star, Inc., Ashland, OR, USA).

## SUPPLEMENTARY MATERIALS FIGURES AND TABLE


