# Supplementary material for: Crosstalk between mismatch repair and base excision repair in human gastric cancer
Source: Oncotarget. 2016 Jun 20;8(49):84827–40. doi: 10.18632/oncotarget.10185 (PMC5689576; doi:10.18632/oncotarget.10185)
Supplement: Supplementary file 1 [file oncotarget-08-84827-s001.pdf]

Crosstalk between mismatch repair and base excision repair in human gastric cancer

SUPPLEMENTARY TABLE AND FIGURES

Supplementary Table S1: Principal component analysis: matrix of component loadings

	Factor Pattern		
**Gene**	**PC1**	**PC2**	**PC3**
apex1	0.49044	0.30584	0.03728
brca1	**0.81696**	-0.33348	-0.06433
brca2	**0.83215**	-0.17682	-0.04684
ercc1	**0.61016**	0.43835	0.17556
fen1	**0.83020**	-0.39551	-0.03008
lig1	**0.78872**	-0.34209	-0.15602
lig3	**0.76870**	0.00052	-0.35560
lig4	**0.58343**	**0.59081**	-0.01517
mbd4	0.54892	0.43910	-0.07017
mlh1	0.39020	**0.51750**	**-0.61112**
mpg	0.22962	0.25156	**0.66689**
mre11a	**0.72541**	0.27787	-0.42487
msh2d	**0.89154**	-0.18232	0.14618
msh3	**0.61222**	0.45434	0.02362
msh6	**0.64816**	**-0.52948**	-0.04180
ogg1	0.31659	0.23467	0.16311
pms2	**0.82304**	0.25019	0.00391
pol β	0.53590	0.15075	**0.69319**
rad51	**0.72403**	-0.45797	0.18433
smug1	0.55300	-0.08195	-0.30781
ung	**0.62546**	-0.44886	0.18908
xpc	0.50154	0.34095	0.37625
xrcc1	0.34676	-0.27068	0.04081

Bolded values identify the most important variables for the determination of component meaning.

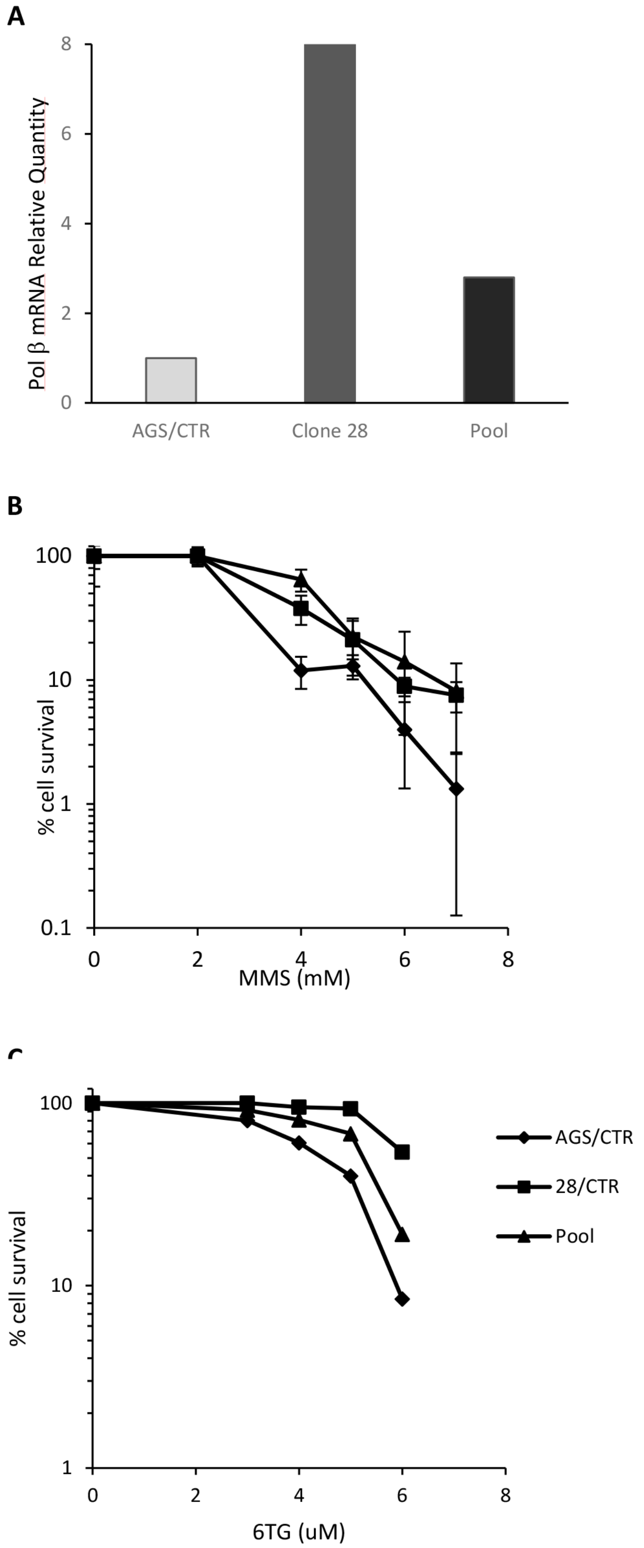

Supplementary Figure S1: Characterization of Polβ over-expressing gastric cancer cells. A. RT-PCR analysis of mRNA from AGS/CTR, clone28 and pooled AGS Polβ over-expressing cells. **B.** Colony survival assay after 2, 4, 5, 6, 7 mM MMS. **C.** Colony survival assay after 3, 4, 5, 6 μM 6-TG.

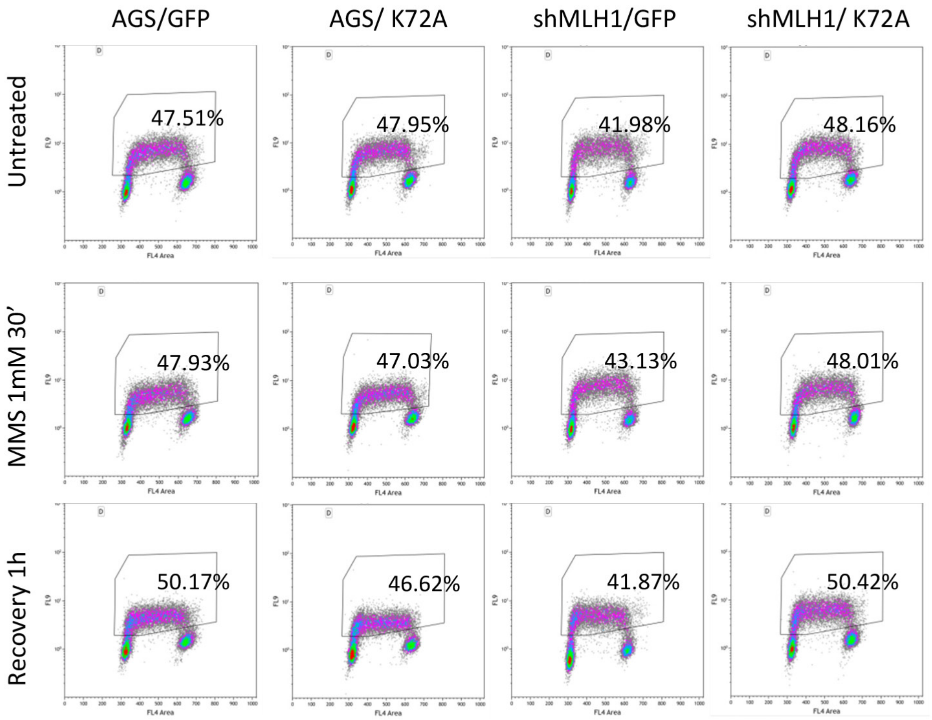

Supplementary Figure S2: Cytofluorimetric bivariate analysis of the MMS-induced cell cycle perturbations in Polβ defective gastric cancer cells with/without inactivation of MLH1. Wild-type (AGS/GFP), Pol β mutant (AGS/K72A), MLH1-deficient (shMLH1/GFP) or Pol β/MLH1-deficient (shMLH1/PolB-K72A) cells were pulse-labeled with BrdU for 30 min to monitor S-phase cells, washed and treated or not with 1 mM MMS for 30 min. At the indicated recovery time, cells were collected and processed for cell cycle analysis as described in Materials and Methods. Cell populations were analysed by gating as reported in the profiles, and the percentage of S-phase cells under each experimental condition is indicated.

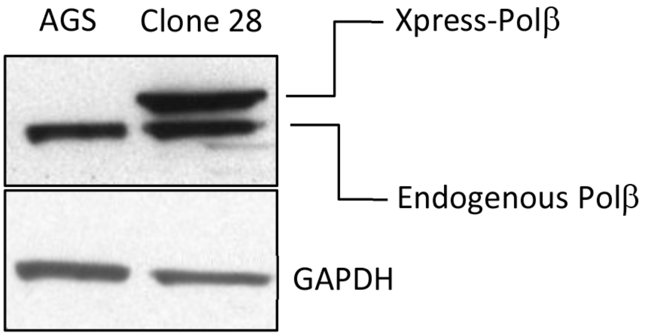

Supplementary Figure S3: Western blot analysis of Polβ in AGS cells and clone 28. Clone 28 is characterized by normal level of endogenous Polβ, compared to AGS cells and by expression of human Polβ Xpress-tagged. GAPDH levels were detected to normalized data.

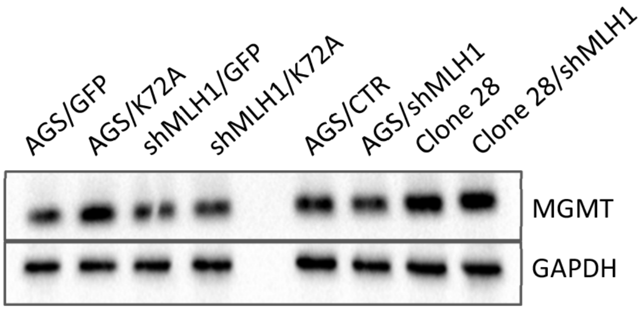

Supplementary Figure S4: Western blot analysis of MGMT in AGS, Polβ over-expressing and Polβ defective gastric cancer cells, with/without inactivation of MLH1. No significant differences were found among levels of MGMT in all cell lines analysed. GAPDH levels were detected to normalized data.
